# The Impact of Coexistence of Smoking and Diabetes on the Coronary Artery Severity and Outcomes following Percutaneous Coronary Intervention: Results from the 1^ST^ Jordanian PCI Registry

**DOI:** 10.1155/2020/7624158

**Published:** 2020-07-02

**Authors:** Mohamad I. Jarrah, Said Al-Khatib, Yousef Khader, Hanin N. AlKharabsheh, Ayman Hammoudeh, Karem H. Alzoubi, Nasr Alrabadi

**Affiliations:** ^1^Department of Internal Medicine, Faculty of Medicine, Jordan University of Science and Technology, Irbid, Jordan; ^2^Department of Physiology, Faculty of Medicine, Jordan University of Science and Technology, Irbid, Jordan; ^3^Department of Public Health, Faculty of Medicine, Jordan University of Science and Technology, Irbid, Jordan 22110; ^4^Cardiology Department, Istishari Hospital, Amman, Jordan; ^5^Department of Clinical Pharmacy, Faculty of Pharmacy, Jordan University of Science and Technology, Irbid, Jordan; ^6^Department of Pharmacology, Faculty of Medicine, Jordan University of Science and Technology, Irbid, Jordan 22110

## Abstract

**Introduction:**

Diabetes mellitus (DM) and smoking are highly prevalent among Middle Eastern patients admitted with acute coronary syndrome (ACS) or who undergo percutaneous coronary intervention (PCI).

**Methods:**

This study used the analysis of the data from the first Jordanian PCI registry (JoPCR1) to determine the impact of coexistence of smoking and diabetes mellitus on the coronary artery severity and outcome following percutaneous coronary intervention in Middle Eastern patients.

**Results:**

Of 2426 patients enrolled, 1300 (53.6%) and 1055 (43.5%) were diabetics and smokers, respectively. The patients' age was 59.0 ± 10.1 and ranged between 24 and 95 years. Males comprised 79.4% of all patients. The patients were divided into four groups: nondiabetic-nonsmokers (22.2%), diabetic-nonsmokers (34.3%), nondiabetic-smokers (24.2%), and diabetic-smokers (19.2%). Compared with the other three groups, patients in the diabetic-nonsmoker group were older, more likely to be females, and having a higher prevalence of hypertension, dyslipidemia, chronic renal disease, and history of CVD and revascularization. Consequently, the diabetic-nonsmoker patients (but not the diabetic-smokers) had a higher prevalence of multivessel CAD and PCI than the other three groups, highlighting the importance of other risk factors (age, gender, metabolic syndrome, and comorbidities) and not only smoking in predisposing for CAD. Furthermore, those patients had a higher incidence of ACS as an indication for PCI than the stable coronary disease (73% vs 27%) and the highest CRUSADE bleeding risk score (63.9%) among other groups. The in-hospital events including in-stent thrombosis and emergency CABG events did not significantly differ among groups (*p* = 0.5 and 0.22). Heart failure and major bleeding events occurred significantly higher among diabetic-nonsmokers compared to other groups. In-hospital deaths occurred significantly more among diabetic-nonsmokers. Moreover, the one-month and one-year follow-up outcome events (the mortality rate, in-stent thrombosis, readmission for ACS, coronary revascularization, and major bleedings) occurred more frequently in the diabetic-nonsmoker group. However, the difference was statistically significant only for major bleeding incidences.

**Conclusions:**

In this analysis of a completed prospective Middle Eastern PCI registry, the majority of the diabetic-nonsmoker (and not the diabetic-smokers) patients (73%) presented with ACS. This group was the highest at risk for in-hospital PCI complications as well as the worst in outcomes after one year of follow-up. Those patients were more likely to be older, female, and have the worst cardiovascular baseline features, highlighting the importance of other risk factors (age, gender, metabolic syndrome, and comorbidities) and not only smoking in predisposing for CAD. Thus, more sufficient education about controlling CVD risk factors should be implemented in the Middle Eastern region.

## 1. Introduction

Coronary artery disease (CAD) is the first of ten leading causes of death worldwide including the Middle East region as noted by the World Health Organization (WHO) [1]. The classical cardiovascular risk factors that contribute to the increase in the morbidity and mortality of CAD include hypertension (HTN), diabetes mellitus (DM), dyslipidemia, and cigarette smoking [2, 3]. It is globally agreed that lifestyle modifications and cardiovascular risk factor control would reduce the likelihood of sustaining acute coronary syndrome. The treatment options for obstructive CAD includes the widely practiced percutaneous coronary interventions (PCI using balloon angioplasty and stent implantation) [4–6]. PCI is commonly used in patients with ST-segment elevation myocardial infarction (STEMI), non-ST-segment elevation myocardial infarction (NSTEMI), or unstable angina (UA). Stent thrombosis is considered the most worrying complication of PCI that carries short- and long-term adverse events [7]. One of the main factors that contribute to the buildup of atherosclerosis is diabetes mellitus (DM), which is a well-known cardiovascular risk factor that adversely affects the coronary vessels. It has been shown to be associated with a 2- to 4-folds increase in the mortality rates for CAD especially after MI and in worsening the patients' long-term prognosis. In addition, diabetic patients who underwent “bare-metal stent” intervention (BMS) or drug-eluting stent (DES) are at high risk for repeating the revascularization procedure during one year of stent implementation [8, 9].

Another preventable high-risk factor for cardiovascular diseases is smoking, which has been shown to have a negative impact on the quality of life of those patients with cardiovascular disease. Cigarettes contain multiple components that negatively influence different organs in the body, such as carbon monoxide (CO), hydrogen cyanide (HCN), nitrogen oxide (NO), acrolein, acetaldehyde, and formaldehyde [10]. Unfortunately, smoking still causes 1 of 5 deaths every year in the United States [11]. It can affect the coronaries with different mechanisms; contributing to the inflammation cascade that triggers the plaque formation in the artery, increasing the level of low-density lipid (LDL), decreasing the level of high-density lipid (HDL), increasing the level of triglyceride, and decreasing the elasticity of the arteries so they become stiff and easily damaged, it may increase the heart rate and consequently the blood pressure, and finally, the carbon monoxide reduces the capacity (affinity) of blood to oxygen [12]. The present study is a post hoc analysis of the first Jordanian PCI registry in Jordanian and other Middle East patients that aimed at determining the impact of the coexistence of DM and smoking on patients who underwent PCI in the short (in hospital) and in the long (one year) outcomes compared to patients who have one or none of these risk factors. The hypothesis was that the coexistence of DM and cigarette smoking will be associated with worse clinical profiles and adverse cardiovascular outcomes.

## 2. Methods

### 2.1. Study Population

The 1^st^ Jordanian PCI registry (JoPCR1) is a prospective multicenter registry of consecutive patients who underwent PCI at 12 tertiary care centers in major cities in Jordan between January 2013 and February 2014 [13]. The study was approved by the Institutional Review Board (IRB) of each participating hospital, and the analysis for the aims of this study has approval from the IRB at King Abdullah University Hospital (780/2018). Informed consents were obtained from enrolled patients, and specific data collecting forms were used to uniform the information obtained from different hospitals during the admission, discharge, and follow-up visits (or phone calls) at 1, 6, and 12 months. The inclusion criteria of the study were adults > 18 years old who were admitted to hospital with ACS or stable coronary disease or ACS and underwent PCI. Patients admitted with stable coronary disease or ACS but treated medically and patients admitted with stable coronary disease or ACS and treated by coronary artery bypass surgery (CABG), as a primary revascularization choice, were excluded from the study.

### 2.2. Data Collection

The collected baseline data for each patient included clinical, laboratory, electrocardiographic, echocardiographic, and coronary angiographic features. A SYNTAX coronary diagram was also used to define the involved arteries and their segments. Details of the PCI procedure and its outcome were also recorded. The arterial access site (radial or femoral), the dual antiplatelet therapy that has been used (aspirin, clopidogrel or ticagrelor, and heparin), and type of stent (drug-eluting (DES), bare-metal (BMS), and left to the operator's discretion) were also obtained; however, DES were used for most of the patients. PCI was indicated for either acute coronary syndrome (ACS) or stable coronary disease (SC). ACS was classified as (1) acute ST-segment elevation MI (STEMI) or (2) non-ST-segment elevation ACS (NSTEACS) and (3) unstable angina (UA).

After PCI implementation, patients were observed in a coronary care or telemetry unit until the time of discharge. During follow-up calls or outpatient clinic visits, almost all patients showed adherence to prescribed medications and incidences of major events were documented. Those major events include death (it was considered that all deaths were cardiac unless it was proven to be a noncardiac cause), admission with ACS, ST, repetition of coronary angiography or revascularization, and pathological confirmation of stent thrombosis by finding recent thrombus within the stent determined at autopsy or by examination of tissue retrieved following thrombectomy.

### 2.3. Statistical Analysis

All statistical analysis was performed using statistical package for social studies (SPSS) software (version 17, Chicago, IL, USA). Continuous variables were expressed as mean ± standard deviation, and statistical differences between groups were accomplished by comparison using Student's unpaired two-sided *t*-test. To assess the correlation between the in-stent thrombosis and the diabetic and smoking as risk factors, we used the chi-square test. Always a *p* value less than 0.05 was considered statistically significant.

## 3. Results

A total of 2426 patients were enrolled in the study with a response rate of 95%. The patients' average age was 59.0 ± 10.1 years, and the age range was between 24 and 95 years old. The percentage of males to females was 79.39% to 20.6%. Patients were divided into four groups: nondiabetic-nonsmokers (22.2%), diabetic-nonsmokers (34.3%), smokers-nondiabetic (24.2%), and diabetic-smokers (19.2%). Of the patients enrolled, 1055 (43.5%) patients were current smokers and 1300 (53%) patients were diabetics.

The baseline features for the diabetic-nonsmokers compared to the other groups are shown in Table 1. This group was the highest in number among the four groups. As well, it contained the highest percentages of females (37.9%), patients with hypertension (76.6%), patients with dyslipidemia (59.3%), patients with chronic renal disease (4.7%%), patients with a history of CVD (46.2%) and coronary revascularization, and patients with low left ventricular ejection fraction (LVEF) (14.3%).

In the clinical presentation (Table 2), we can see that the percentages of patients presented with ACS, stable coronary disease, and one or multivessel diseased were higher in the diabetic-nonsmoker group. As well, the need for revascularization with PCI, crusade bleeding risk score for predicting the major bleeding event, and the mortality prediction grace risk score were the highest among the diabetic-nonsmoker group. On the other hand, only the STEMI occurs more with one of the other groups (the nondiabetic-smoker group).

The in-hospital complications in diabetic smokers compared with the 3 groups are shown in Table 3. Diabetic-nonsmokers were significantly the highest in heart failure (*p* value = 0.023) and in-hospital deaths (*p* value = 0.014). There was a trend of increase in the major hospital bleeding events within this group as well (*p* value = 0.07).

Finally, Table 4 shows the results of follow-up for one and 12 months after stent implantation. The follow-up evaluation focuses on cardiovascular events in diabetic smokers compared with the 3 groups. Although relatively high incidences were reported within the diabetic-nonsmokers' group, those differences were not significant.

## 4. Discussion

### 4.1. Overview and Summary of Results

Coronary artery disease is one of the major health concerns in terms of morbidity and mortality all around the world. Atherosclerosis and its associated CAD are the leading cause of ischemic heart diseases such as myocardial infarction, heart failure, arrhythmias, and sudden cardiac death [14]. The prognosis of the patients depends on the amount of myocardium lost as a result of the ischemia/reperfusion injury [14]. Without doubt, timely reperfusion by the thrombolytic or percutaneous coronary intervention (PCI) can salvage ischemic myocardium and, indeed, can restore the normal perfusion in the coronaries [15].

Several risk factors for CAD facilitate the incidence of ischemic heart disease and myocardial infarction. DM and smoking usually ranked for the worst to be able to cause a significant effect on the CAD especially among Middle Eastern (ME) patients, either in their baseline characteristic as a cause for the disease or in terms of in-hospital and posttreatment with PCI complications with up to one-year follow-up [16, 17].

For that, we designed this study to explore the impact of those two major strong risk factors, on the severity of coronary artery disease and the therapeutic outcomes following PCI in Middle Eastern patients.

In summary, we found that about 34.3% of the Middle Eastern patients were diabetic and nonsmokers; the majority of them (73%) were admitted with ACS. The diabetic-nonsmoker group may be considered the worst at baseline cardiovascular risk profile, having more severe CAD; possesses the highest needs for multivessel PCI, highest GRACE risk score, and highest CRUSADE bleeding risk score at discharge, compared with the other 3 groups; and was at higher risk for in-hospital events. Finally, we found that PCI complications and one-year postdischarge events occur more frequently in the diabetic-nonsmoker group.

### 4.2. Diabetes and Its Correlation with CAD

Diabetes mellitus has a strong association with coronary artery disease, especially in older patients [18]. In parallel to our diabetic-nonsmoker baseline findings, others found that diabetics had worse baseline cardiovascular risk profile compared with nondiabetic patients.

Several unique characteristics of the Middle Eastern population are included in the present study. The high prevalence of diabetes is alarming as diabetes is accounted for about half of the patients undergoing PCI, and this result is consistent with previous studies on the Jordanian population [19].

Compared to other registries in the west, the prevalence of diabetes does not exceed (25-35%) and this rate is much lower than those of Middle Eastern ACS population [20].

Furthermore, the current finding regarding the diabetic-nonsmoker group agreed with other ACS registries in the region [21, 22]. It indicated that our patients are more likely to be females, older, and suffering from dyslipidemia, hypertension, chronic renal disease, and history of CAD or treated with PCI and CABG. Moreover, diabetes prevalence, smoking status, and obesity may all reflect the poor social, physical activity, and exercise, as well as the poor healthy type of food that we have in our region [23]. Diabetes, by virtue of myriad processes, is associated with severe atherothrombotic CAD [24, 25].

The deleterious cardiovascular outcome of diabetics observed within patients treated with PCI or medically by thrombolytic, related to several known pathological and clinical factors associated with diabetes [26, 27]. Diabetic patients tend to have longer complex lesions, small coronary artery size, high on-treatment platelet reactivity, with an accelerated unstable disease, and of higher risk for in-stent thrombosis and revascularization [28, 29].

Those previous findings supported our results and indicated that diabetics had the worst cardiovascular profile. The PCI population in our study group consisted of four-fifth of ACS patients and one-fifth with a stable coronary syndrome, which was higher with ACS compared to western studies where the ACS patients represent two-third of the population [22, 30]. This difference can be confirmed by other epidemiological studies where they indicated that the cardiac population tends to present with more acute complications than the stable disease in the developing countries compared to western countries [31, 32].

The in-hospital event and one-year follow-up outcomes, in diabetic patients of our study, were higher than the nondiabetic; stent thrombosis, readmission with ACS, the need for revascularization, and major bleeding events, however, were still lower than what other registries reported [6, 33].

The main unexpected finding in our study was that the diabetic-nonsmokers were at high risk for cardiovascular events compared to the diabetic-smokers. In recent studies where they investigated the effect of smoking cessation in a Middle Eastern population and post PCI procedures, they reported that smoking quitters were more likely to have diabetes, low EF, and heart failure compared with persistent smokers [34]. Undoubtedly, nicotine is the most effective long-term drug for controlling weight over the past century, but the delivery route of nicotine among people by smoking cigarettes makes it extraordinarily toxic because it is combined with a toxic component of the cigarette [35]. Moreover, such an explanation may be questionable especially that the BMI for the diabetic-smoker group was higher than that for the diabetic-nonsmoker group. On the other hand, lipid profile appeared to be more uncontrolled among the diabetic-nonsmokers. LDL level > 100 mg/100 ml (bad lipids) occurs more frequently in diabetic-nonsmokers, HDL (the good lipids) was lower, and the triglyceride level was found to be higher. This was again in contrast to other studies, where the LDL level increased parallel to the increase in VLDL and proportionally with smoking. As well, patients with chronic kidney disease are seen much more in the diabetic-nonsmoker group. It is well known that diabetes is a leading cause of chronic kidney diseases (microvascular diseases), but in concordance to smoking status, the diabetic-smoker group in our study has much less patient with kidney disease. In our group of diabetic-nonsmokers, we should acknowledge that they have a higher prevalence of hypertension which in turn may affect the kidney's function regardless of the smoking status. Moreover, diabetic-nonsmokers who underwent PCI are older than their counterparts in the other groups. The younger age is expected to give a good prognosis, and PCI will give excellent results compared with elderly patients with PCI [36]. While our study surprisingly showed that the diabetic-nonsmokers have the worst outcomes compared to the diabetic-smokers, we showed as well that this group of patients was the worst when considering other risk factors for CAD. Smoking cessation ranked among the most powerful strategies to prevent CAD, and it is strongly advocated by the treatment guidelines of patients with ACS [37]. As we previously mentioned before about smoking and its harmful effect on multiorgan diseases, comprehensive effort programs for smoking cessation must be extended beyond patient discharge from the hospital.

Current findings do not favor the patients to smoke if they were diabetics; smoking and DM are still considered major risk factors that have a critical role in CAD. Tight control on DM patients, lifestyle modifications, increase awareness about healthy food, physical activity, and smoking cessation will improve the coronary vessel, and there outcome posts PCI implementation. Smoking cessation with other acceptable behavioral and therapeutic methods should be followed by patients to modulate CAD risk factors. Education and proper physical consultations are warranted to our patients in the Middle Eastern region.

### 4.3. Limitations and Recommendations

This was a nonrandomized comparison of four different groups of patients; thus, it cannot be free from the inherent limitations of observational registries such as allocation bias and collection of nonrandomized data. Although there was a prospective enrolment of consecutive patients, participation was voluntary and the inclusion of all comers was not verified. Besides, patients with ACS who died before or shortly after admission and those who did not undergo angiography or PCI were not represented in this study.

Cigarette smoking was dealt with as all or none risk factors. Further analysis should evaluate cardiovascular outcomes according to the amount and duration of smoking. Furthermore, we did not evaluate other forms of smoking prevalence in our society, especially Argile smoking. The participating hospitals are high volume tertiary care centers; thus, the results cannot be generalized to the whole region. We need to confirm these results in a larger number of patients enrolled not only in tertiary care hospitals but also in community hospitals and to include those who smoke other forms than cigarettes.

## 5. Conclusion

In this analysis of a completed prospective Middle Eastern PCI registry, diabetic-nonsmoker patients accounted for about one-third of the PCI population. The majority of them (73%) presented with ACS, more likely to be older, female, and have the worse cardiovascular baseline features with a higher risk for in-hospital PCI complications as well as the worst outcome up to one year of follow-up. Those results can highlight the importance of other risk factors (age, gender, metabolic syndrome, and comorbidities) and not only smoking in predisposing for CAD. Comprehensive tight control on DM and other cardiovascular risk factors are not less important than secondary effective strategies for smoking cessation that should be extended beyond hospital discharge and should be implemented to improve cardiovascular outcomes.

## Figures and Tables

**Table 1:** Baseline features of the diabetic-smokers compared with the other 3 groups in a cohort of 2426 patients.

Clinical variable	Total number	Diabetic-smokers	Diabetic-nonsmokers	Nondiabetic-smokers	Nondiabetic-nonsmokers	*p* value
Number (%)	2426	467 (19.2%)	833 (34.3%)	588 (24.2%)	538 (22.2%)	<0.0001
Females (*n*, %)	500	32 (6.9%)	316 (37.9%)	31 (5.3%)	121 (22.5%)	<0.0001
Hypertension (*n*, %)	1511	299 (64.0%)	638 (76.6%)	247 (42.0%)	327 (60.8%)	<0.0001
≤45 years of age	308	83 (17.8%)	34 (4.1%)	158 (26.9%)	33 (6.1%)	<0.0001
Dyslipidemia (*n*, %)	1184	247 (52.9%)	494 (59.3%)	221 (37.6%)	222 (41.3%)	<0.0001
BMI ≥ 25 kg/m^2^	1877	430 (92%)	661 (79.4%)	422 (71.8%)	364 (67.7%)	0.003
Chronic renal disease	69	10 (2.1%)	39 (4.7%)	6 (1.0%)	14 (2.6%)	0.001
Family history of premature CVD	956	221 (47.3%)	301 (36.1%)	241 (41%)	193 (35.9%)	<0.0001
Past history of CVD	954	180 (38.5%)	385 (46.2%)	178 (30.3%)	211 (39.2%%)	0.0001
Past history of coronary revascularization (PCI or CABG)	673	123/PCI 16/CABG = 139 29.7%	230/PCI 30/CABG = 260 31.2%	106/PCI 14/CABG = 120 20.4%	130/PCI 24/CABG = 154 28.6%	Pci:0.0001 CABG:0.528
Low LVEF (≤45%)	302	62 (13.3%)	119 (14.3%)	64 (10.9%)	57 (10.6%)	0.123

BMI: body mass index; CABG: coronary artery bypass graft; CVD: cardiovascular disease; LVEF: left ventricular ejection fraction; PCI: percutaneous coronary intervention; SD: standard deviation (*p* ≤ 0.05, significant).

**Table 2:** Clinical presentation, coronary artery disease, and PCI indications in diabetic-smokers compared with the other 3 groups.

Clinical variable	Diabetic-smokers	Diabetic-nonsmokers	Nondiabetic-smokers	Nondiabetic-nonsmokers	*p* value
Total number	467	833	588	538	
Presentation:					0.0001
ACS (*n*, %)	380 (81.4%)	610 (73.2%)	482 (82%)	398 (74%)	
Stable coronary disease (*n*, %)	87 (22.9%)	223 (26.8%)	106 (18%)	140 (26%)	
STEMI (*n*, %)	165 (35.3%)	184 (22.1%)	238 (40.5%)	139 (25.8%)	0.0001
NSTEMI (*n*, %)	66 (14.13%)	85 (10.2%)	92 (15.6%)	63 (11.7%)	
Multivessel CAD (*n*, %)	195 (41.8%)	385 (46.2%)	211 (35.9%)	218 (40.5%)	0.001
Multivessel PCI (*n*, %)	128 (27.4%)	279 (33.5%)	137 (23.3%)	144 (26.8%)	0.018
High GRACE risk score at discharge (highest tertile)	123 (26.3%)	353 (42.3%)	125 (21.3%)	230 (42.9%)	0.008
CRUSADE bleeding risk score (highest 2 pentile)	132 (28.6%)	533 (63.9%)	90 (15.3%)	189 (35.2%)	0.0001

ACS: acute coronary syndrome; CAD: coronary artery disease; GRACE risk score; CRUSADE bleeding risk score; STEMI: ST-segment elevation myocardial infarction.

**Table 3:** In-hospital complications in diabetic-smokers compared with the other 3 groups.

Event	Diabetic-smokers	Diabetic-nonsmokers	Nondiabetic-smokers	Nondiabetic-nonsmokers	*p* value
Ventricular arrhythmia	4 (0.9%)	6 (0.7%)	4 (0.7%)	7 (1.3%)	0.863
Heart failure	35 (7.5%)	84 (10.1%)	45 (7.7%)	30 (5.6%)	0.023
Stent thrombosis	1 (0.2%)	5 (0.6%)	2 (0.3%)	1 (0.2%)	0.501
Emergency CABG	0 (0%)	0 (0%)	1 (0.2%)	2 (0.4%)	0.222
Major bleeding	3 (0.6%)	14 (1.7%)	0 (0%)	6 (1.1%)	0.07
In-hospital death	3 (0.6%)	13 (1.6%)	2 (0.3%)	1 (0.2%)	0.014

**Table 4:** One- and 12-month cardiovascular events in diabetic-smokers compared with the 3 groups.

Clinical variable	Diabetic-smokers	Diabetic-nonsmokers	Nondiabetic-smokers	Nondiabetic-nonsmokers	*p* value
One-month events (from admission to one month):					
Mortality	4 (0.9%)	16 (1.9%)	4 (0.7%)	5 (0.9%)	0.121
Stent thrombosis	4 (0.9%)	11 (1.3%)	6 (1%)	3 (0.6%)	0.559
Readmission for ACS	7 (1.5%)	22 (2.6%)	11 (1.9%)	5 (0.9%)	0.128
Coronary revascularization	5 (1.1%)	13 (1.6%)	8 (1.4%)	4 (0.7%)	0.579
Major bleeding	3 (0.6%)	15 (1.8%)	3 (0.05%)	6 (1.1%)	0.249
12-month events:					
All-cause mortality	9 (1.9%)	29 (3.5%)	9 (1.5%)	11 (2%)	0.626
Stent thrombosis	13 (2.8%)	28 (3.4%)	17 (2.9%)	12 (2.2%)	0.152
Readmission for ACS	14 (3%)	35 (4.2%)	16 (2.7%)	13 (2.4%)	0.623
Coronary revascularization	12 (2.6%)	19 (2.3%)	10 (1.7%)	7 (1.3%)	0.159
Major bleeding	11 (2.4%)	29 (3.5%)	6 (1%)	4 (0.7%)	0.046

## Data Availability

The data used to support the findings of this study are available from the corresponding author upon request.
